# Rotational Angiography 3D Data Set to Confirm Harmony Valve Fitness Post Pulmonary Artery Plication

**DOI:** 10.1016/j.jscai.2024.102518

**Published:** 2025-01-13

**Authors:** Osamah Aldoss, Thomas Panicucci, Bassel Mohammad Nijres, Mohsen Karimi

**Affiliations:** a Pediatric Cardiology, Stead Family Children’s Hospital, University of Iowa, Iowa City, Iowa; b Pediatric Cardiothoracic Surgery, Stead Family Children’s Hospital, University of Iowa, Iowa City, Iowa

**Keywords:** fit analysis, hybrid, rotational angiography, transcatheter pulmonary valve

## Abstract

A 28-year-old woman presented with heart failure symptoms secondary to severe pulmonary valve regurgitation in the setting of congenital pulmonary valve stenosis that required balloon valvuloplasty. Computed tomography angiography fit analysis demonstrated a severely dilated main pulmonary artery that did not meet the minimum oversizing criteria for placement of a 25-mm Harmony transcatheter pulmonary valve. A hybrid transcatheter pulmonary valve placement approach took place, and a post main pulmonary artery plication right ventricle rotational angiogram was performed. The patient underwent a successful transcatheter pulmonary valve placement. The 3D rotational angiography data set was analyzed, and fit analysis demonstrated an adequate oversizing of the 25-mm transcatheter pulmonary valve.

## Clinical case

**Figure 1:**
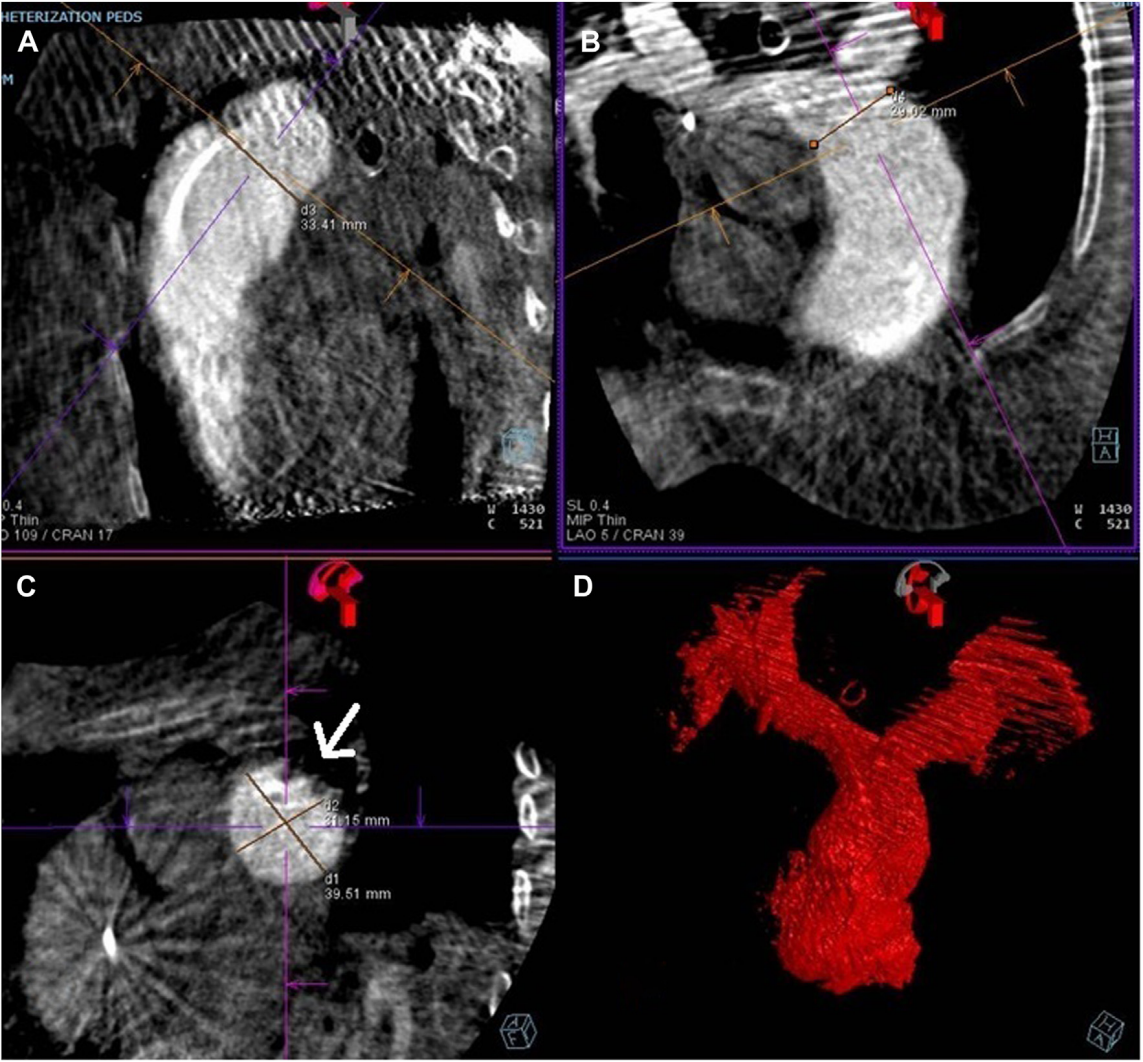
**Multiplanar reformat data from the rotational angiography showing the reduction in distal MPA size (A,B, and C).** The site of plication is seen in (C) as indicated by the arrow. (D) shows the 3D model created from the 3DRA data set. 3DRA, 3D rotational angiography; MPA, main pulmonary artery.

**Figure 2:**
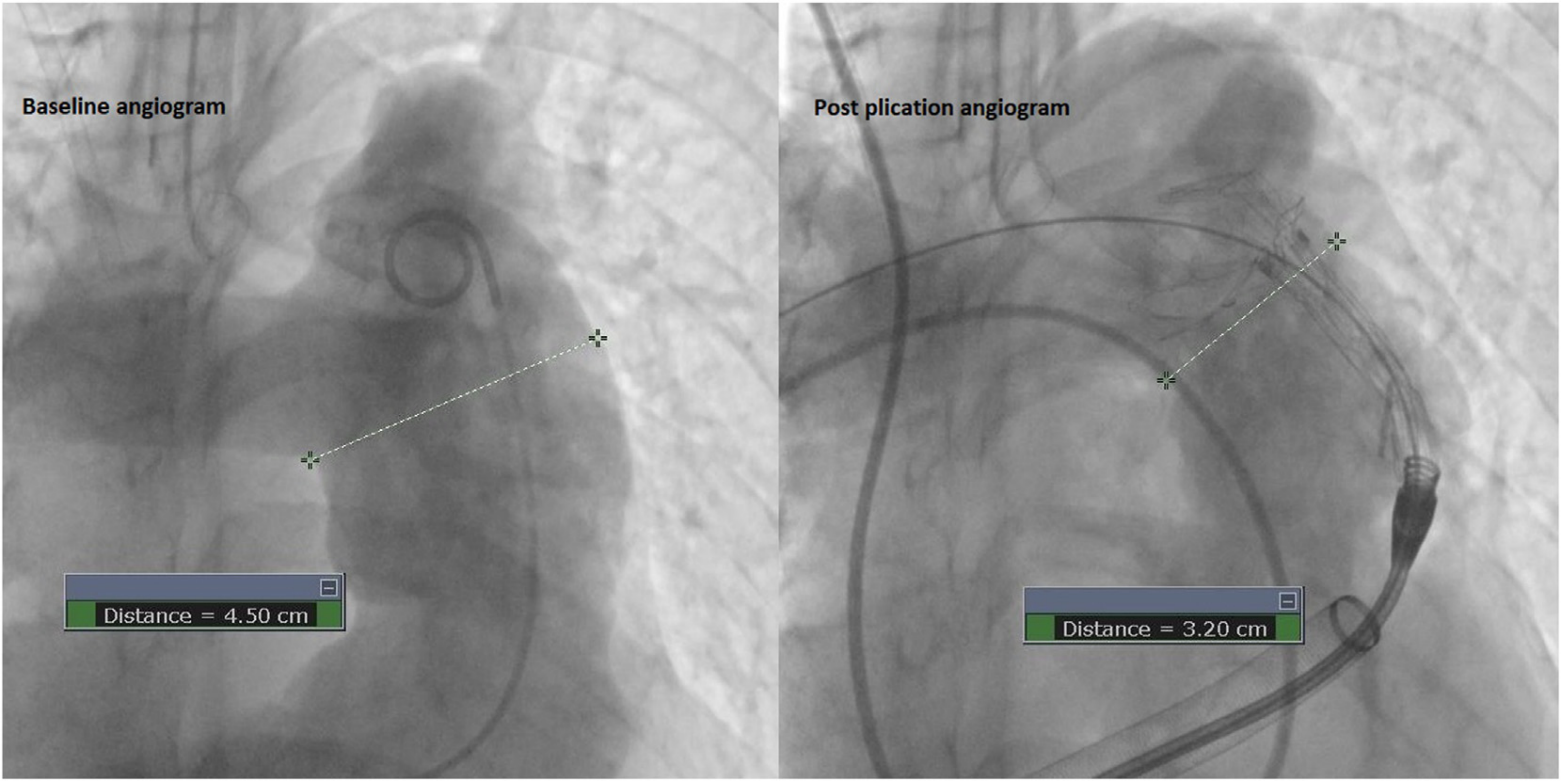
**Baseline and post plication AP angiogram showing the difference in measurements at the distal MPA segment.** AP, anteroposterior; MPA, main pulmonary artery.

**Figure 3:**
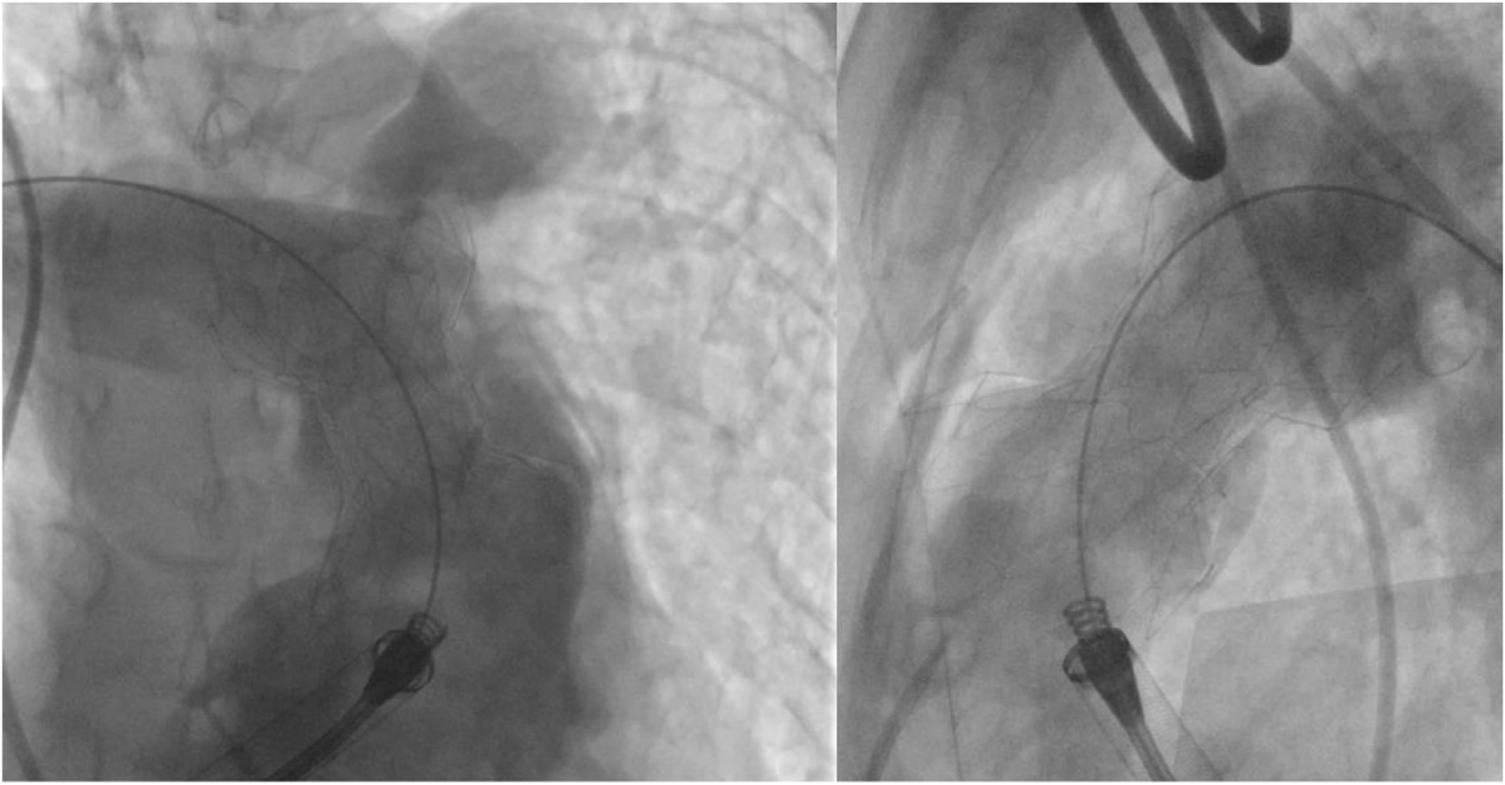
**Post valve placement right ventricle angiogram showing a well seated Harmony valve with good interference of the distal MPA.** MPA, main pulmonary artery.

**Figure 4:**
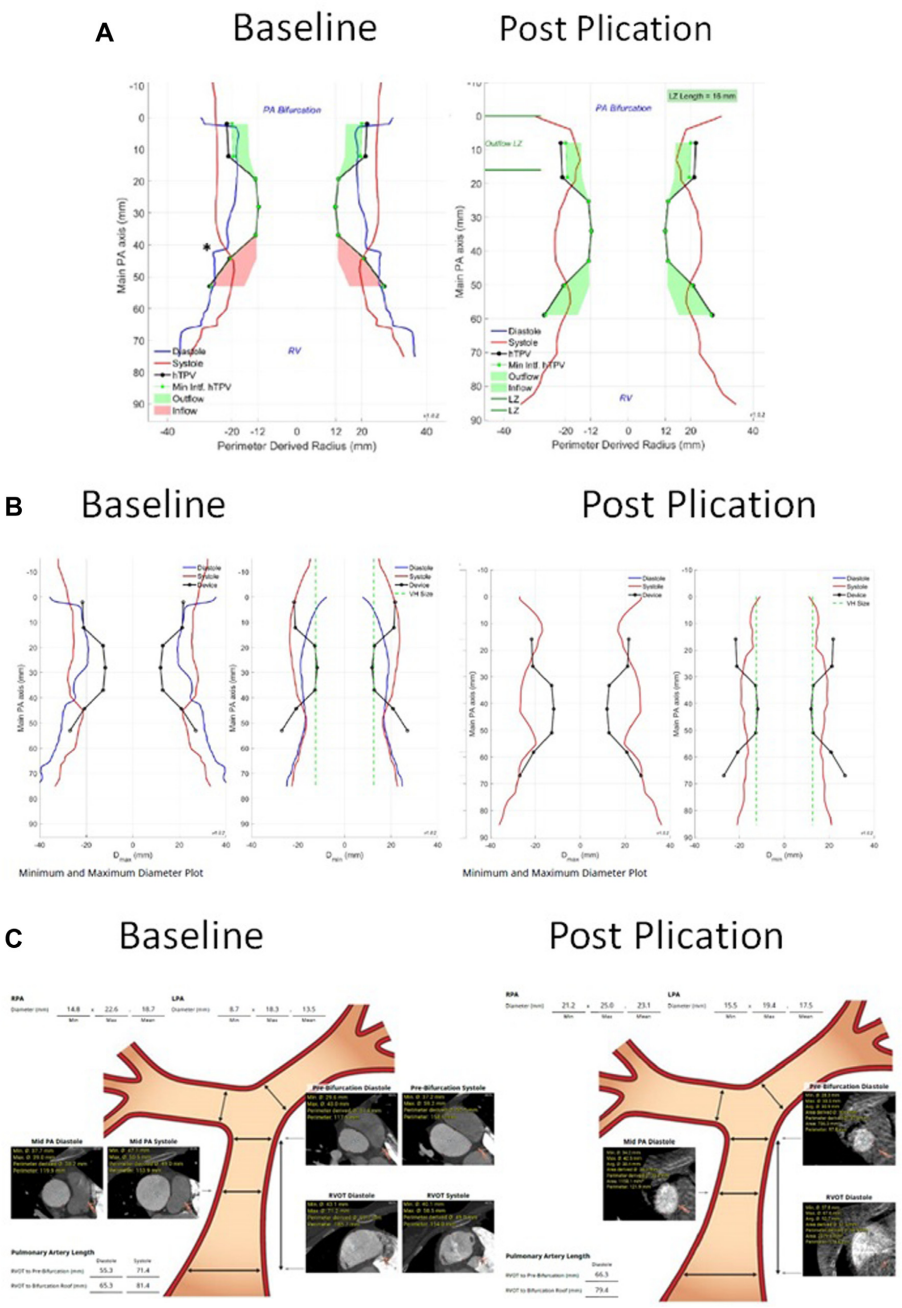
(A) Perimeter-derived radius comparison between baseline and post plication showing an improved simultaneous adequate oversizing of both device ends. (B) Minimum and maximum diameter plot at baseline and post plication that clearly shows a reduction in the distal MPA diameter. (C) Cross sectional diameters at different levels showing a reduction in size at the distal MPA. Baseline images from preprocedural CTA and post plication images from the 3D data set from the rotational angiogram. CTA, computed tomography angiogram; MPA, main pulmonary artery.

A 28-year-old woman presented with a history of congenital pulmonary valve stenosis post balloon valvuloplasty shortly after birth. The patient presented with heart failure symptoms in the setting of severe pulmonary valve regurgitation and severe dilated right ventricle (RV). Computed tomography angiography fit analysis demonstrated a severely dilated main pulmonary artery (MPA) that did not meet minimum oversizing criteria to safely place a 25-mm Harmony transcatheter pulmonary valve (TPV) (Medtronic). Options including surgical and off-label hybrid approaches for valve placement were discussed with the patient. The patient decided to proceed with the hybrid approach, which included surgical MPA plication via left anterior thoracotomy followed by TPV placement. As described in a previous publication,^1^ the procedure took place in the hybrid catheterization laboratory, followed by right heart catheterization and then securing a stiff wire in the right lower pulmonary artery. Next, surgical MPA plication via left anterior thoracotomy took place and was followed by RV rotational angiogram. The 3-dimensional rotational angiography data set was analyzed in the catheterization laboratory to assess changes in the anatomy. There was adequate modification of the anatomy, including a significant reduction in size at the distal MPA as seen on the multiplanar reformat and 3D model (Figure 1). Based on these findings, we proceeded with TPV, and the angiograms confirmed the adequacy of the anatomic modification (Figure 2). The patient underwent a successful TPV (Figure 3) with a trivial para-valvar leak on the immediate intracardiac echocardiogram. A formal fit analysis was performed after the procedure by Medtronic confirmed an adequate and simultaneous oversizing of both ends of the 25-mm Harmony TPV (Figure 4). At 6 months follow-up, the patient is doing well with improved symptoms and better exercise tolerance. Her echocardiogram showed a well-functioning valve including Doppler assessment with peak gradient of 24 mm Hg, mean gradient of 14 mm Hg, and trivial para-valve leak.

Pulmonary artery plication using a hybrid approach to modify the RV outflow tract (RVOT) for TPV has been described.^1^^,^^2^ Two-dimensional angiography and compliant balloon testing post plication were used to assess the suitability of the anatomy for valve placement. The left anterior thoracotomy approach to modify the RVOT led to a noncircumferential reduction in RVOT size. The rotational angiogram provided a 3D understanding of the RVOT, which allowed for better assessment of device candidacy. One limitation with the 3D rotational angiography data set from the rotational angiogram is the ungated nature and need to average over the cardiac cycle, which can lead to a suboptimal analysis. Nonetheless, this provided additional data to confirm the adequacy in size reduction and provided reassurance for device stability (Figures 1 and 4).
